# Lifestyle mediates the association between normal-weight visceral obesity and MASLD in older adults with hypertension and diabetes

**DOI:** 10.3389/fnut.2026.1845959

**Published:** 2026-08-28

**Authors:** Jun-Yan Xi, Yi-Jing Wang, Xiao-Heng Li, Rui-Qi Ming, Hua-Ling Yan, Xue-Qi Li, Jie Sun, Wei Hu, Jie Hu, Bo Yan, Jian-Jun Bai, Yi-Ning Xiang, Jing Gu, Xiao Lin, Gang Liu, Yuan-Tao Hao

**Affiliations:** 1 Department of Medical Statistics, School of Public Health, Sun Yat-sen University, Guangzhou, Guangdong, China; 2 Sun Yat-sen Global Health Institute, Sun Yat-sen University, Guangzhou, Guangdong, China; 3 Center for Health Information Research, Sun Yat-sen University, Guangzhou, Guangdong, China; 4 Shenzhen Center for Disease Control and Prevention, Shenzhen, China; 5 Department of Epidemiology and Health Statistics, School of Public Health, Guangdong Medical University, Dongguan, China; 6 Chengdu Integrated TCM&Western Medicine Hospital (Chengdu First People's Hospital), Chengdu, Sichuan, China; 7 Institute for the Control of Infectious Diseases, Guizhou Center for Disease Control and Prevention, Guiyang, Guizhou, China; 8 Department of Epidemiology, School of Public Health, Sun Yat-sen University, Guangzhou, Guangdong, China; 9 Department of Maternal, Child and Adolescent Health, School of Public Health, Tongji Medical College, Huazhong University of Science and Technology, Wuhan, Hubei, China; 10 Guangzhou Medical University Library, Guangzhou, Guangdong, China; 11 Department of Epidemiology & Biostatistics, School of Public Health, Peking University, Beijing, China; 12 Peking University Center for Public Health and Epidemic Preparedness & Response, Beijing, China; 13 Key Laboratory of Epidemiology of Major Diseases, Peking University, Ministry of Education, Beijing, China

**Keywords:** causal inference models, cohort studies, healthy lifestyle, mediating effects, metabolic dysfunction-associated steatotic liver disease, multimorbidity, older adults

## Abstract

**Objective:**

Older adults with coexisting hypertension and type 2 diabetes are at high risk for metabolic dysfunction-associated steatotic liver disease (MASLD). Normal-weight visceral obesity (NWVO)—excess visceral fat despite normal body mass index and waist circumference—is an overlooked risk factor. Lifestyle is modifiable, but its role in explaining the NWVO-MASLD association is unknown. We quantified the proportion of this association statistically mediated by unhealthy lifestyle patterns.

**Methods:**

This retrospective cohort study included 26,289 adults aged ≥50 years with hypertension and type 2 diabetes from the Shenzhen National Basic Public Health Services Program (2010–2023). NWVO was defined using the Chinese Visceral Adiposity Index alongside normal body size criteria. MASLD was diagnosed by ultrasound and cardiometabolic criteria. A composite lifestyle score (physical activity, alcohol, smoking, dietary salt intake) was derived. We applied the generalized front-door formula for causal mediation analysis to estimate the path-specific effect of NWVO on MASLD incidence explained by lifestyle, robust to unmeasured confounding.

**Results:**

Over a mean 5.5-year follow-up, NWVO was associated with a significantly increased MASLD risk (adjusted *HR*: 1.82, 95% *CI*: 1.76, 1.89). In marginal models, NWVO was associated with a 2.35-fold higher risk of having an unhealthy lifestyle (2.25, 2.44) and an 11.2% higher MASLD risk (8.7, 13.8). Using the front-door formula, unhealthy lifestyle statistically explained 19.8% (12.1, 33.6) of the total NWVO-MASLD effect, corresponding to a mediated risk increase of 41.2% (3.5, 92.7).

**Conclusion:**

In this multimorbid older population, NWVO is a significant MASLD risk factor, and modifiable lifestyle patterns account for approximately one-fifth of this association. Our findings support integrating visceral adiposity assessment into routine care and implementing multifaceted lifestyle interventions—targeting diet, physical activity, and substance use—to reduce the burden of MASLD in aging populations with metabolic comorbidities.

## Introduction

Metabolic dysfunction-associated steatotic liver disease (MASLD) imposes a substantial burden on older adults with comorbid hypertension and type 2 diabetes, a growing population worldwide (1–5). Normal-weight visceral obesity (NWVO)—excess visceral adiposity despite normal body mass index and waist circumference—is an increasingly recognized but frequently overlooked risk factor in this group (6–8). Visceral adipose tissue drives hepatic steatosis through the release of free fatty acids and pro-inflammatory mediators, while unhealthy lifestyle behaviors independently promote fat accumulation and liver injury (9–13). However, the extent to which NWVO increases MASLD susceptibility in older adults already affected by hypertension and diabetes remains unclear, particularly given that over 40% of global MASLD cases occur in non-obese individuals (14).

Identifying modifiable pathways connecting NWVO to MASLD in this population is essential for designing preventive strategies. Lifestyle is a promising candidate because of its well-documented effects on liver health and its modifiability (15). However, conventional mediation analysis is invalidated by unmeasured confounding between NWVO and MASLD, inherent to their shared metabolic and environmental determinants (16, 17). To address this, we employed the generalized front-door formula within a structural causal model framework (18, 19). In this analysis, lifestyle is modeled as a statistical mediator rather than a strict temporal mediator. In particular, the goal is not to establish a unidirectional causal chain, but to quantify the proportion of the NWVO–MASLD association that is statistically explained by differences in lifestyle patterns. This reframes lifestyle as a modifiable explanatory factor, even though biological relationships among visceral adiposity, behaviors, and liver disease are likely bidirectional and complex.

Using a large longitudinal cohort of older adults with hypertension and type 2 diabetes in South China, we aimed to (1) assess whether NWVO confers an elevated risk of developing MASLD, and (2) estimate the proportion of this association statistically mediated by unhealthy lifestyle. These findings provide evidence to inform lifestyle-targeted strategies for reducing the MASLD burden in this vulnerable population.

## Methods

### Study design and participants

**Figure 1:**
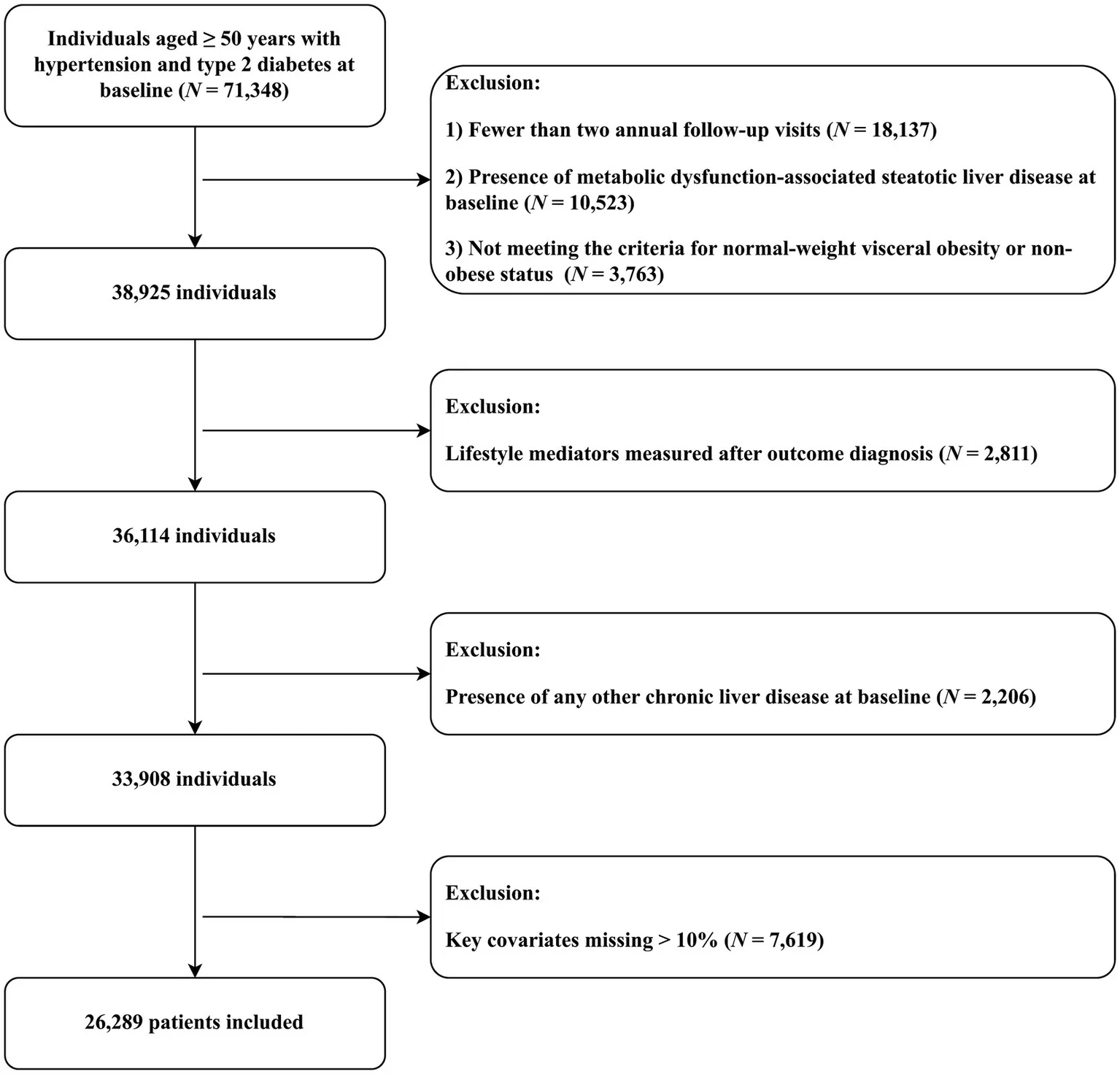
Flow chart of patient recruitment.

This cohort study employed data from the National Basic Public Health Services Program (NBPHSP) of Shenzhen, China. The NBPHSP, which forms an integral part of China’s Universal Health Coverage, aims to establish an integrated health service system based on primary health care (20). Shenzhen, a megacity located in southern China, is globally known for its rapid and advanced reforms to the primary healthcare system (21). A total of 71,348 individuals aged 50 years and above who were enrolled in the NBPHSP in Shenzhen between 2010 and 2023 were selected for inclusion in the study. All participants were diagnosed with hypertension and type 2 diabetes at the primary health-care institutions, and disease-specific health records and regular follow-ups (including demographic characteristics, life behaviors, and clinical examination) were established. Inclusion criteria in this study were (1) aged ≥50 years with a comorbid diagnosis of hypertension and type 2 diabetes at baseline; (2) completion of at least two follow-up visits per year on average; (3) absence of medical imaging evidence or clinical diagnosis of MASLD at baseline; (4) classification as non-obese based on Chinese criteria (BMI < 24 kg/m^2^ and waist circumference < 90 cm for males or < 85 cm for females), allowing subsequent categorization into NWVO and non-obese reference groups based on CVAI levels. The exclusion criteria were (1) measurement of lifestyle mediator variables occurred after the diagnosis of MASLD; (2) missing data for > 10% of the key covariates. The final dataset employed in the analysis comprised 26,289 participants (Figure 1).

The scientific protocol and operational procedures of this study were approved by the Ethics Committee at Shenzhen Center for Disease Control and Prevention, and all participants provided written informed consent before their involvement.

### NWVO as exposure

NWVO was defined using a two-step approach. First, to identify visceral adiposity dysfunction, we used the Chinese Visceral Adiposity Index (CVAI). The CVAI is an appropriate index for the evaluation of visceral fat dysfunction in the Chinese population (22). In brief, CVAI integrates age, body mass index, waist circumference, triglycerides, and high-density lipoprotein cholesterol into a sex-specific equation (Appendix Text S1). We selected CVAI because it has been validated against abdominal computed tomography in Chinese adults and its components are routinely collected in primary care settings, making it a practical tool for large-scale epidemiological screening when direct imaging of visceral fat is not feasible. Although CVAI includes triglycerides and high-density lipoprotein cholesterol—which are also components of the MASLD cardiometabolic diagnostic criteria—this does not constitute circular inference. Our study used a prospective cohort design. Specifically, CVAI was assessed only at baseline solely for the purpose of classifying exposure status, while the outcome, MASLD, was ascertained during subsequent follow-up visits. Thus, the exposure definition and outcome diagnosis are temporally and analytically separated. This component overlap could make the observed association between NWVO and MASLD appear somewhat stronger than it truly is. However, our primary question of how much of this association is statistically explained by lifestyle is less affected because this question concerns the share of the association attributable to lifestyle rather than the total magnitude of the association itself. A validated cutoff of 79.19 for predicting MASLD risk in Chinese adults, derived from the independent Jinchang cohort, was applied to classify participants into “high CVAI” (≥79.19) and “low CVAI” (<79.19) groups (10). Second, to isolate the normal-weight phenotype, we excluded any participants with abnormal body size according to Chinese criteria (BMI ≥ 24 kg/m^2^ and/or waist circumference ≥90 cm for males or ≥85 cm for females). This resulted in two groups, the NWVO group (high CVAI with normal body size) and the non-obese reference group (low CVAI with normal body size). The use of an externally validated cutoff ensures that the exposure definition is not influenced by outcome data from the current study, thereby avoiding the potential for circular inference.

### Unhealthy lifestyle as mediator

Information on lifestyle was obtained from participants during the baseline and follow-up periods. The frequency of each lifestyle factor was quantified based on the number of times participants met the pre-specified effective criteria per week or month, as detailed in Appendix Text S2. Lifestyle was assessed across four distinct dimensions: (1) exercise frequency, defined as daily, regular weekly, occasionally, or never; (2) alcohol consumption, defined as never, occasionally, often, or daily; (3) smoking status, denoted as never, ever, or current; and (4) salt intake, classified as low or high. A lifestyle-weighted score was then developed to evaluate their combined impact on MASLD risk (9) (Appendix Text S3). Using marginal structural models to balance potential confounders, we estimated the independent association (*β* coefficient, the natural logarithm of the hazard ratio) between each level of each lifestyle factor and MASLD incidence (Appendix Table S1). The weighted score for each individual was calculated as the sum of the corresponding *β* coefficients across the four dimensions, yielding a continuous composite score ranging from 0 to 3.861 across 144 possible combinations, with higher scores indicating a more stringent healthy lifestyle. The *β* coefficients were used exclusively to derive the weighted score and its dichotomization threshold. These coefficients were not incorporated into the front-door mediation analysis, which treats the binary lifestyle classification as the mediator and estimates the path-specific effect independently. Thus, the score derivation and the causal mediation are analytically distinct steps, and the front-door estimation does not reuse the *β* coefficients. When stratified by quartiles, individuals with scores exceeding the 75th percentile (i.e., >2.323) exhibited a greater than 90% reduction in MASLD risk relative to the lowest quartile (Appendix Table S2), confirming a strong protective threshold. Accordingly, we defined an unhealthy lifestyle as a weighted score ≤2.323 (≤75th percentile). To preserve the temporal sequence essential for causal mediation analysis, we ensured that the lifestyle-weighted score used for each participant was always ascertained from assessments conducted before the diagnosis of MASLD.

### MASLD as outcome

The primary diagnosis of MASLD was based on the presence of steatotic liver disease, as confirmed by transabdominal ultrasound, and we also referred to any one of the cardiometabolic criteria outlined as outlined in Appendix Text S4 for further confirmation of MASLD (5). Participants in this study had comorbid hypertension and type 2 diabetes. Both conditions are cardiometabolic criteria for diagnosing MASLD, and therefore hepatic steatosis in this population could be diagnosed as MASLD (9).

### Statistical analyses

The directed acyclic graph was employed to identify relevant covariates, including sex, age group, education, marriage, follow-up results for hypertension and diabetes, and drug compliance for hypertension and diabetes (23) (Appendix Figure S1). To initially assess the association between baseline NWVO status and the time-to-onset of MASLD, we constructed a Cox proportional hazards model. Time-varying covariates were updated at each follow-up visit and incorporated into the model as time-dependent covariates. The Cox model relies on the proportional hazards (PH) assumption, which posits that the hazard ratio for any covariate is constant over time. We rigorously evaluated this assumption for all variables. We rigorously evaluated this assumption by testing the statistical significance of interaction terms between each covariate and the logarithm of follow-up time. A statistically significant interaction (*p* < 0.05) indicates that the effect of the covariate on the hazard changes over time, thereby violating the PH assumption. For any covariate that violated the PH assumption, the model was extended by including a time-dependent interaction term, defined as the product of the covariate and a function of time. This adjustment allows the hazard ratio to vary over time, thereby resolving the violation and yielding unbiased effect estimates.

The primary causal effect of interest is the path-specific effect, i.e., the effect of NWVO on the risk of developing MASLD that is mediated by an unhealthy lifestyle. We used the generalized front-door formula to estimate this mediated effect within a structural causal model framework, following previous studies (18, 19). The generalized front-door formula relies on three structural conditions: (1) the mediator (unhealthy lifestyle) intercepts the paths from exposure to outcome that are influenced by unmeasured confounders; (2) measured covariates sufficiently account for confounding of the exposure–mediator relationship; and (3) conditional on the exposure and measured covariates, there is no unmeasured confounding of the mediator–outcome relationship. In our setting, condition (1) is supported by the breadth of the lifestyle-weighted score, which integrates four behavioral domains that are established determinants of both visceral adiposity and MASLD and thus may serve as a conduit for upstream influences. Conditions (2) and (3) are supported by the rich covariate set available in the NBPHSP and by the well-characterized direct effects of lifestyle on MASLD risk. A formal articulation of these conditions and their justification is provided in Appendix Text S5. The analysis proceeded in four key steps: (1) First, we fitted a time-dependent Cox proportional hazards model (the exposure-mediator model) for the effect of the exposure on the mediator, adjusting for the aforementioned covariates. Concurrently, a second time-dependent Cox model (the mediator-outcome model) was fitted for the effect of the mediator on the outcome, adjusting for the same set of covariates and the exposure. These models were fitted to inform the subsequent simulations. (2) Second, simulated mediators were predicted using the empirical parameters derived from the exposure-mediator model and a simulated exposure variable that was rendered marginally independent of the confounders. Specifically, this simulated exposure was constructed by pooling two datasets: one in which all individuals’ exposures were set to “no” and another in which all were set to “yes.” (3) Third, simulated outcomes were predicted using the empirical parameters from the mediator-outcome model, the original observed exposures, and the newly simulated mediators. (4) Finally, a marginal time-dependent Cox proportional hazards model was constructed using the simulated exposures and outcomes to derive a point estimate of the causal contrast, which represents the path-specific front-door effect in this study. Steps in the generalized form of the front-door formula are provided in Appendix Text S6 and Appendix Figure S2. Conceptually, this analysis positions lifestyle as a statistical mediator, but not as a strict temporal mediator. While lifestyle factors may biologically precede or co-evolve with visceral adiposity, our analytic objective is to quantify the extent to which the observed NWVO-MASLD association is statistically explained by differences in lifestyle patterns between groups. The generalized front-door formula decomposes the total association into a component operating through lifestyle and a component operating through other pathways. This interpretation reframes lifestyle as a modifiable explanatory factor—a key intervention target—rather than positing a strict unidirectional temporal chain from NWVO to lifestyle to MASLD. Such an approach is particularly appropriate when the NWVO and lifestyle may be interrelated through complex bidirectional or confounding relationships that are not fully captured by traditional mediation assumptions.

Multiple imputation by chained equations was used to handle sporadic missing values among the 26,289 participants, and the primary analyses were conducted on the imputed datasets (24). Variable-specific missingness is reported in Appendix Table S3. In addition, we conducted conventional mediation analysis using the R package *mediation* to assess the robustness of the results from the generalized front-door formula (25).

### Sensitivity analyses

To assess the robustness of our primary findings, we conducted several sensitivity analyses using alternative analytical strategies and definitions: (1) to evaluate the potential impact of the exposure definition, the primary analysis was repeated using an alternative CVAI cutoff (131.35) derived from a data-driven approach (maximally selected rank statistics) within the current study cohort (26, 27) (Appendix Text S7 and Appendix Figure S3). This exploratory cutoff was used to test whether the main findings were sensitive to the choice of threshold. (2) The dichotomization threshold for unhealthy lifestyle was altered from the 75th to the 50th percentile of the lifestyle-weighted score to examine whether the mediation effect was sensitive to the chosen cutoff. (3) To evaluate whether the mediating role of lifestyle differed by sex, we conducted sex-stratified analyses using the generalized front-door formula. The path-specific front-door effect was estimated separately for males and females. (4) A complete-case analysis was conducted by excluding all participants with any missing data in key covariates to assess potential bias introduced by multiple imputation. (5) To minimize potential exposure misclassification from changes in body composition over time, a subgroup analysis was restricted to participants whose NWVO status remained unchanged from baseline throughout the entire follow-up period.

## Results

### Characteristics of the participants in Shenzhen from 2010 to 2023

Compared to the reference group, the exposure group had a higher proportion of females (89.9% vs. 29.4%) and individuals aged 70 years or older (70 to 79 years: 39.4% vs. 19.8%; 80 to 89 years: 11.5% vs. 2.5%; ≥90 years: 0.8% vs. 0.1%). They also had higher rates of only primary school education (38.5% vs. 24.2%), illiteracy or semiliteracy (9.4% vs. 3.1%), widowhood (8.9% vs. 2.4%), and divorce (0.5% vs. 0.3%). Additionally, the exposure group exhibited only slightly lower mean BMI (22.26 vs. 22.77) and slightly higher mean levels of triglycerides (1.66 mmol/L vs. 1.30 mmol/L) and high-density lipoprotein cholesterol (1.32 mmol/L vs. 1.26 mmol/L). In contrast, the mean CVAI was markedly higher in the exposure group (143.72 vs. 105.72) (Table 1).

**Table 1:** Statistical description of characteristics of the participants in Shenzhen, China, from 2010 to 2023.

Variable	Overall	Reference group	Exposure group	*p*-value
Number of participants	26,289	20,909	5,380	
Sex (%)				<0.001
Males	15,314 (58.3)	14,770 (70.6)	544 (10.1)	
Females	10,975 (41.7)	6,139 (29.4)	4,836 (89.9)	
Age group (%)				<0.001
50 to 59 years	2,370 (9.0)	2,261 (10.8)	109 (2.0)	
60 to 69 years	16,448 (62.6)	13,963 (66.8)	2,485 (46.2)	
70 to 79 years	6,251 (23.8)	4,130 (19.8)	2,121 (39.4)	
80 to 89 years	1,148 (4.4)	528 (2.5)	620 (11.5)	
90 years and older	72 (0.3)	27 (0.1)	45 (0.8)	
Education (%)				<0.001
College or above	4,379 (16.7)	3,774 (18.0)	605 (11.2)	
High school	6,970 (26.5)	5,945 (28.4)	1,025 (19.1)	
Second school	6,662 (25.3)	5,485 (26.2)	1,177 (21.9)	
Primary school	7,120 (27.1)	5,051 (24.2)	2069 (38.5)	
Illiterate or semiliterate	1,158 (4.4)	654 (3.1)	504 (9.4)	
Marriage (%)				0.021
Never	17 (0.1)	14 (0.1)	3 (0.1)	
Married	25,203 (95.9)	20,332 (97.2)	4,871 (90.5)	
Widowed	977 (3.7)	499 (2.4)	478 (8.9)	
Divorce	92 (0.3)	64 (0.3)	28 (0.5)	
BMI (mean, SD)	22.66 (1.97)	22.77 (2.06)	22.26 (1.47)	<0.001
Waist, cm (mean, SD)	83.19 (5.80)	83.20 (5.56)	83.17 (6.64)	0.715
TG, mmol/L (mean, SD)	1.38 (0.55)	1.30 (0.53)	1.66 (0.57)	<0.001
HDL-C, mmol/L (mean, SD)	1.27 (0.26)	1.26 (0.26)	1.32 (0.27)	<0.001
CVAI (mean, SD)	113.49 (24.18)	105.72 (20.31)	143.72 (10.19)	<0.001
Follow-up result for hypertension (%)				0.404
Satisfactory control	24,808 (94.4)	19,718 (94.3)	5,090 (94.6)	
Unsatisfactory control	1,481 (5.6)	1,191 (5.7)	290 (5.4)	
Follow-up result for diabetes (%)				<0.001
Satisfactory control	22,051 (83.9)	17,656 (84.4)	4,395 (81.7)	
Unsatisfactory control	4,238 (16.1)	3,253 (15.6)	985 (18.3)	
Drug compliance for hypertension (%)				0.940
Regularly	23,802 (90.5)	18,935 (90.6)	4,867 (90.5)	
Intermittently	940 (3.6)	749 (3.6)	191 (3.6)	
Not taking medicine	1,547 (5.9)	1,225 (5.9)	322 (6.0)	
Drug compliance for diabetes (%)				0.014
Regularly	23,486 (89.3)	18,737 (89.6)	4,749 (88.3)	
Intermittently	833 (3.2)	630 (3.0)	203 (3.8)	
Not taking medicine	1723 (6.6)	1,347 (6.4)	376 (7.0)	
No medication required	247 (0.9)	195 (0.9)	52 (1.0)	

BMI, Body mass index; TG, Triglyceride; HDL-C, High-density lipoprotein cholesterol; CVAI, Chinese visceral adiposity index; SD, Standard deviation.

### Lifestyle of the participants

Table 2 illustrates that, in comparison to the reference group, the exposure group exhibited a lower prevalence of daily (54.9% vs. 56.4%) or regular weekly (18.4% vs. 21.5%) exercise. Additionally, the exposure group had markedly lower proportions of never-drinkers (80.4% vs. 87.0%) and never-smokers (72.9% vs. 89.6%), coupled with a higher prevalence of high-salt intake (54.5% vs. 52.4%).

**Table 2:** Statistical description of the lifestyle of the participants in Shenzhen, China, from 2010 to 2023.

Variable	Overall	Reference group	Exposure group	*p*-value
Number of participants	26,289	20,909	5,380	
Exercise frequency (%)				<0.001
Daily	14,755 (56.1)	11,802 (56.4)	2,953 (54.9)	
Regular weekly	5,490 (20.9)	4,501 (21.5)	989 (18.4)	
Occasionally	2,256 (8.6)	1770 (8.5)	486 (9.0)	
Never	3,788 (14.4)	2,836 (13.6)	952 (17.7)	
Alcohol consumption (%)				<0.001
Never	22,520 (85.7)	18,197 (87.0)	4,323 (80.4)	
Occasionally	2,449 (9.3)	1987 (9.5)	462 (8.6)	
Often	615 (2.3)	585 (2.8)	30 (0.6)	
Everyday	705 (2.7)	140 (0.7)	565 (10.5)	
Smoking status (%)				<0.001
Never	22,667 (86.2)	18,742 (89.6)	3,925 (72.9)	
Ever	1768 (6.7)	1,259 (6.0)	509 (9.5)	
Current	1854 (7.1)	908 (4.3)	946 (17.6)	
Salt intake (%)				0.005
Low	13,887 (52.8)	9,956 (47.6)	2,446 (45.5)	
High	12,402 (47.2)	10,953 (52.4)	2,934 (54.5)	

### Follow-up time and risk of MASLD in the exposure group

**Figure 2:**
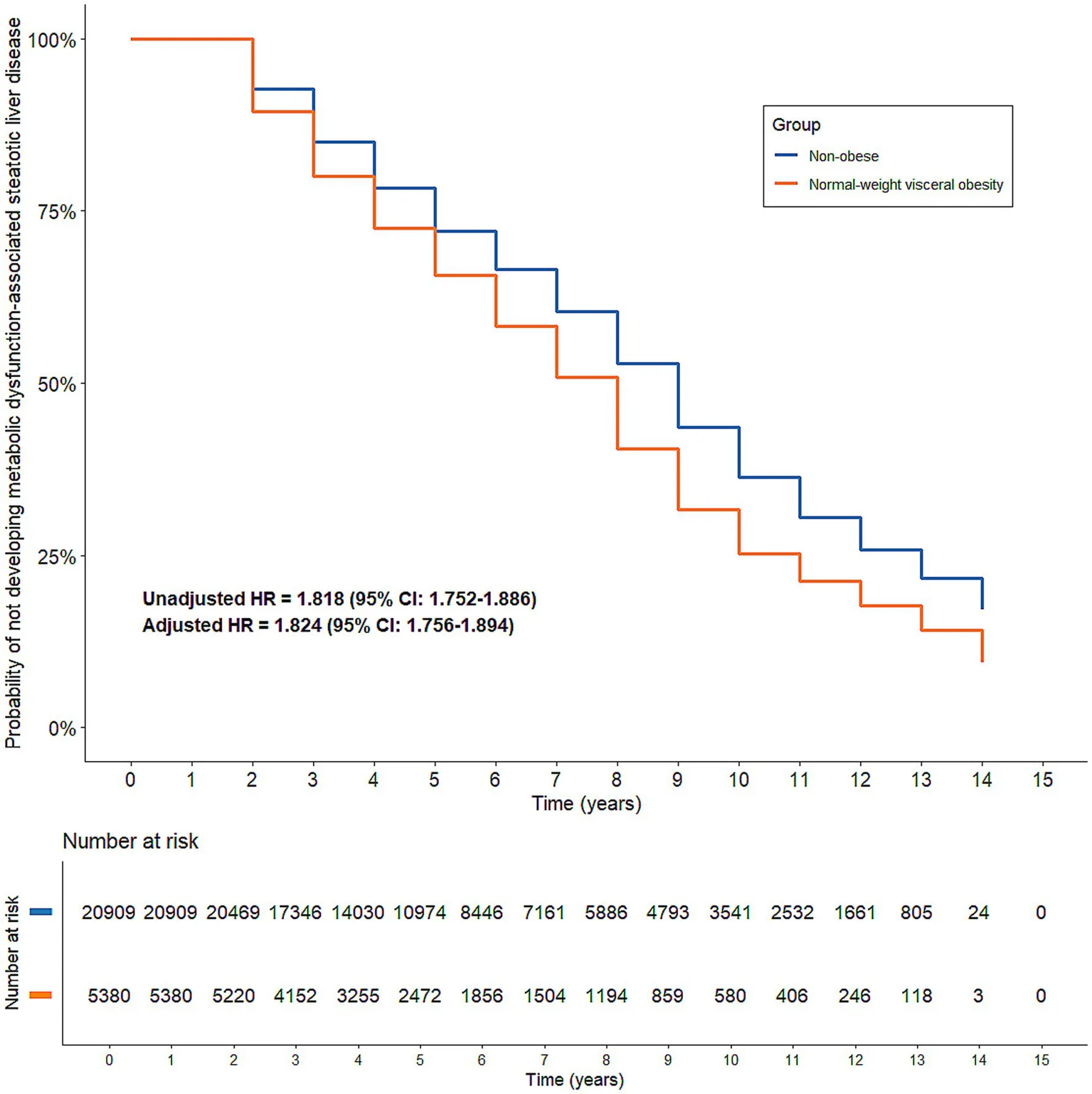
Follow-up time and the risk of MASLD in the exposure group. HR, Hazard ratio; CI, Confidence interval.

During a mean follow-up of 5.5 years (totaling 145,822 person-years), 11,791 participants developed MASLD. Specifically, the reference group accumulated 118,577 person-years of follow-up, during which 9,184 events occurred, yielding an incidence rate of 77.5 per 1,000 person-years (95% *CI*: 75.9, 79.1). The exposure group accumulated 27,245 person-years of follow-up and experienced 2,607 events, corresponding to an incidence rate of 95.7 per 1,000 person-years (95% *CI*: 92.0, 99.4). In the Cox proportional hazards regression analysis, adjusted for covariates identified by the directed acyclic graph, the risk of MASLD was significantly higher in the exposed group than in the reference group (adjusted *HR*: 1.824, 95% *CI*: 1.756, 1.894) (Figure 2).

### Effect estimates for causal paths of exposure to mediator and mediator to outcome

**Figure 3:**
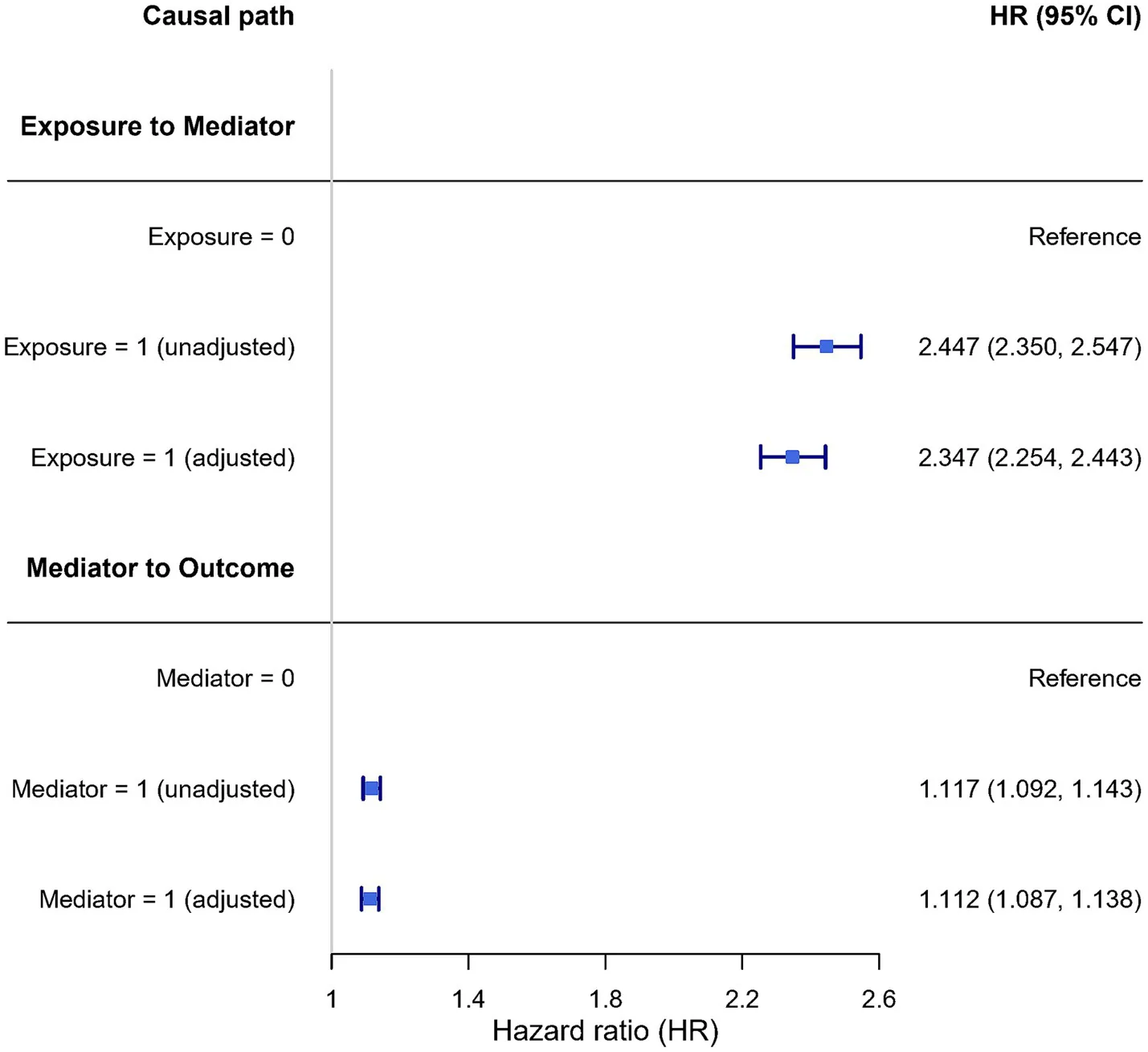
Effect estimates for the exposure-to-mediator and mediator-to-outcome paths. CI, confidence interval. Exposure is NWVO, mediator is the healthy lifestyle, and outcome is metabolic dysfunction-associated steatotic liver disease.

A noteworthy correlation was identified between exposure and mediation, with the proportion of individuals who adopted an unhealthy lifestyle among those with NWVO demonstrating a 2.347 times (adjusted *HR*: 2.347, 95% *CI*: 2.254, 2.443) increase compared to those with non-obesity. Similarly, an unhealthy lifestyle was significantly associated with the outcome, with an 11.2% increase in MASLD risk (adjusted *HR*: 1.112, 95% *CI*: 1.087, 1.138). While this effect size is modest at the individual level, it translates to a meaningful population-level impact given the high prevalence of unhealthy lifestyle in the NWVO group. The effect estimates in the remaining models without covariate adjustment remained largely consistent (Figure 3).

### Causal effect from exposure to outcome through mediator

**Figure 4:**
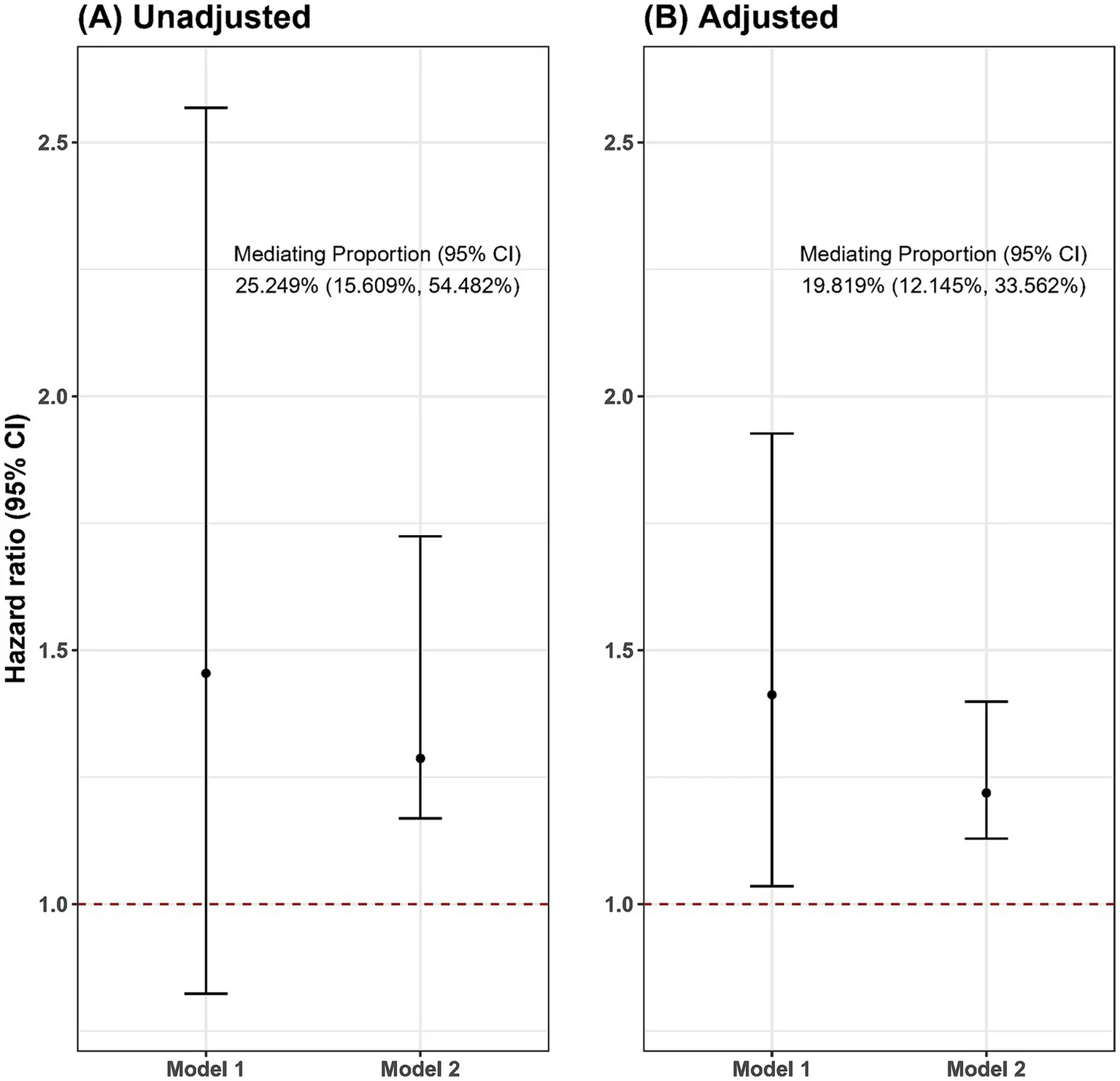
Causal effect from exposure to outcome through mediator. CI, Confidence Interval. Model 1 is the generalized front-door formula, and Model 2 is conventional mediation analysis using the R package *mediation*. Panel **A** shows the unadjusted estimates, while Panel **B** shows the adjusted estimates, with covariates identified by the directed acyclic graph.

The results of the generalized front-door formula demonstrated that a meaningful portion of the NWVO-MASLD association was statistically attributable to differences in lifestyle. Specifically, the risk increment statistically explained by unhealthy lifestyle was 41.2% (adjusted *HR*: 1.412, 95% *CI*: 1.035, 1.927), corresponding to a proportion of the total association explained of 19.819% (95% *CI*: 12.145, 33.562%) (Figure 4, Model 1). The estimated natural indirect effect from conventional mediation analysis (adjusted *HR*: 1.219, 95% *CI*: 1.129, 1.399) was slightly smaller in magnitude than the path-specific front-door effect derived from the generalized front-door formula (Figure 4, Model 2).

### Sensitivity analyses

Sensitivity analyses demonstrated the robustness of our primary findings (Appendix Table S4). Using an alternative data-driven cutoff (131.35) yielded consistent estimates, with a significant natural indirect effect (adjusted *HR*: 1.298, 95% *CI*: 1.021, 1.651) and a mediated proportion of 16.452% (95% *CI*: 10.123, 28.665). Changing the lifestyle mediator cutoff to the 50th percentile attenuated the path effects but maintained a significant indirect effect (adjusted *HR*: 1.205, 95% *CI*: 1.073, 1.353) and a stable proportion mediated (18.151%; 95% *CI*: 11.251, 30.112). Among females, the indirect effect through unhealthy lifestyle remained significant (adjusted *HR*: 1.451; 95% *CI*: 1.078, 1.953), with a mediating proportion of 21.034% (95% *CI*: 12.487, 35.624). Among males, the point estimate was directionally consistent but accompanied by wide confidence (adjusted *HR*: 1.318; 95% *CI*: 0.892, 1.957; mediating proportion: 16.758%; 95% *CI*: 7.234, 34.521), reflecting the limited number of males with NWVO. Complete-case analysis produced point estimates similar to those from the primary imputed analysis, albeit with wider confidence intervals (adjusted *HR*: 1.409, 95% *CI*: 1.023, 1.942). In the subgroup with stable NWVO status, the indirect effect was strengthened (adjusted *HR*: 1.501, 95% *CI*: 1.152, 1.956), with a mediated proportion of 22.448% (95% *CI*: 14.912, 36.812). Collectively, these analyses confirm that the mediating role of lifestyle between NWVO and MASLD risk is robust across different definitions and analytical assumptions.

## Discussion

In this large-scale longitudinal cohort of 26,289 older adults in southern China with comorbid hypertension and type 2 diabetes, we found a significantly elevated risk of MASLD associated with NWVO, a frequently overlooked yet consequential risk factor. Using the generalized front-door model to address intractable unmeasured confounding, we delineated that this heightened risk was meaningfully mediated by modifiable lifestyle factors. This finding has considerable clinical relevance as individuals with NWVO often remain undetected and unaware of their metabolic risk due to the absence of overt obesity signs, potentially delaying intervention until advanced disease stages. Our study moves beyond association by applying a causal inference framework to support the mediating role of lifestyle. We estimated that approximately one-fifth of the effect of NWVO on MASLD incidence in this high-risk population was mediated through unhealthy lifestyle patterns. Given the global prevalence of hypertension, diabetes, and NWVO as comorbid patterns in aging societies, these insights offer supporting evidence for refined healthy aging strategies. Specifically, our study may support incorporating NWVO screening into the routine clinical management of older adults with this high-risk comorbidity profile to guide targeted lifestyle interventions and reduce MASLD burden.

### NWVO is associated with an unhealthy lifestyle

One of our findings is that individuals with NWVO may be deficient in adopting a healthy lifestyle. It has been demonstrated that visceral adipose dysfunction represents a significant contributing factor to the development of metabolic disorders (28–30). However, the conventional indicators employed to evaluate obesity are inadequate for assessing this particular form of body fat accumulation, which is not readily discernible (31). One possible explanation is that the absence of overt obesity in NWVO may reduce both the individual’s perception of metabolic risk and the clinician’s vigilance in recommending lifestyle changes. And yet, further study may still be warranted for validation of these mechanisms. Previous studies in children and young adults have shown that self-perceived weight status can influence health behaviors (32, 33), but whether this mechanism operates in older adults with comorbidities remains untested. Our findings—that NWVO is associated with a higher prevalence of unhealthy lifestyle—are consistent with this hypothesis, but direct measures of risk perception and health awareness may be needed to confirm it.

### An unhealthy lifestyle is linked to an increased risk of developing MASLD

Although the individual-level effect size was modest, the high prevalence of unhealthy lifestyle in the NWVO group amplifies its population-level relevance, supporting the rationale for broad lifestyle interventions. This finding was also corroborated in the general population, where a lifestyle is typically gauged by tobacco and alcohol consumption, dietary habits, physical activity, and sleep patterns (34–39). Our results provide further evidence using a causal inference approach among older adults with comorbidities. In particular, our study supports the aforementioned finding that never smoking or never drinking may confer greater benefits in reducing risk (9). Furthermore, we revealed that a strict healthy lifestyle, as defined by a lifestyle-weighted score above the 75th percentile, confers substantial health benefits in reducing the risk of developing MASLD among older adults with comorbidities. These findings demonstrate the necessity of implementing intervention strategies and the efficacy of adhering to a combination of healthy lifestyles to prevent the development of MASLD.

### From NWVO to the risk of developing MASLD through an unhealthy lifestyle

We observed that the association between NWVO and the risk of developing MASLD in comorbid older adults is partially mediated by unhealthy lifestyle. Although the body mass index and waist circumference of individuals are within the normal range, visceral adipose dysfunction may be associated with an increased risk of developing MASLD. The mediating role of lifestyle observed in our study is consistent with evidence from other settings where behavioral factors were shown to partially explain health disparities (40–43). However, the mediated proportion in our study was approximately one-fifth and was larger than that typically reported for socioeconomic or early-life exposures. More specifically, previous studies have identified visceral adiposity as a major driver of MASLD risk. Its effect may be partly mediated by metabolic factors influenced by lifestyle behaviors (44, 45). Emerging evidence on normal-weight obesity suggests that individuals with this phenotype have metabolic profiles and health behaviors that differ from those of lean and overtly obese individuals. These findings further support the role of lifestyle as a potential mediator between visceral adiposity and liver outcomes (6, 46, 47). Improving unhealthy lifestyle behaviors may contribute to reducing the comorbidity burden of MASLD among older adults with NWVO. Last but not least, given the substantial mediated proportion, additional interventions combining lifestyle modification and pharmacotherapy may be warranted for older adults with NWVO and comorbidities. (48).

### Strengths and limitations

The principal strength of this study is the utilization of the generalized front-door formula. This approach recovers unbiased path-specific estimates in the presence of unmeasured exposure–outcome confounding, which is particularly advantageous given the shared metabolic and genetic architecture of NWVO and MASLD. An important conceptual perspective is that lifestyle was modeled as a statistical mediator. Specifically, the analysis quantified the proportion of the NWVO–MASLD association explained by lifestyle differences, without assuming a unidirectional temporal chain. This supports the clinical rationale for lifestyle interventions even though biological relationships are likely bidirectional.

We acknowledge several limitations. First, both exposure and outcome relied on surrogate indices. NWVO was defined using CVAI rather than direct imaging, while MASLD was diagnosed by ultrasound, which has limited sensitivity for mild steatosis. Under the assumption of non-differential misclassification, our estimates may be conservative, although validation data are needed in the future to determine the exact magnitude. Second, while our primary analysis employed an externally validated CVAI cutoff to avoid circular inference, the choice of cutoff remains a potential source of uncertainty in the main effect estimates. The alternative cutoff derived from a data-driven approach within this cohort produced similar effect estimates, albeit with slightly different magnitudes. This suggests that our findings are not highly sensitive to the specific threshold. Third, despite adjustment for covariates identified using the directed acyclic graph, residual confounding may remain. Unmeasured factors, including socioeconomic characteristics, dietary quality, medication classes, and genetic traits, may confound the exposure–mediator or mediator–outcome paths and are not addressed by the front-door formula (49–52). And yet, the stability of the mediated proportion across sensitivity analyses provides indirect evidence of robustness. Fourth, this single-city study in Shenzhen may not generalize to rural or less-developed settings. However, the finding that lifestyle is a meaningful mediator likely holds across populations even if the precise proportion varies. Additionally, the NWVO group comprised predominantly older females, consistent with the known epidemiology of this phenotype (53, 54). Our findings are therefore most directly applicable to this subgroup. Fifth, while CVAI-based screening is low-cost, formal implementation studies are needed to establish feasibility and cost-effectiveness at scale.

## Conclusion

This study demonstrates that NWVO significantly drives the development of MASLD among older adults with comorbid hypertension and type 2 diabetes—a high-risk group representing a growing clinical challenge worldwide. Our findings establish that lifestyle factors mediate a substantial proportion of this risk and provide robust causal evidence to support targeted interventional strategies within this complex multimorbidity context. These results strongly advocate for the integration of visceral adiposity assessment into standardized clinical practices to identify high-risk individuals who would most benefit from multifaceted lifestyle interventions. Given that the CVAI relies on measurements already collected in routine chronic disease management, such integration could be implemented at modest incremental cost, though formal implementation studies are needed to confirm feasibility and cost-effectiveness at scale. Such interventions have the potential to reduce a significant proportion of MASLD cases, thereby alleviating the multifaceted burden of this disease in aging societies.

## Data Availability

The original contributions presented in the study are included in the article/Supplementary material, further inquiries can be directed to the corresponding authors.
