# Supplementary material for: Lifestyle mediates the association between normal-weight visceral obesity and MASLD in older adults with hypertension and diabetes
Source: Front Nutr. 2026 Aug 28;13:1845959. doi: 10.3389/fnut.2026.1845959 (PMC13564254; doi:10.3389/fnut.2026.1845959)
Supplement: Supplementary file 1 [file Data_Sheet_1.PDF]

**Lifestyle Mediates the Association Between Normal-Weight Visceral Obesity and MASLD in Older Adults with Hypertension and Diabetes**

# Appendix

Appendix Text S1. Development of CVAI ........ 2
Appendix Text S2. Collection for lifestyle variables ........ 3
Appendix Text S3. Weighted score for the four lifestyles ........ 4
Appendix Text S4. Diagnostic criteria of MASLD........ 5
Appendix Text S5. Assumptions of the generalized front-door formula and their justification ........ 6
Appendix Text S6. Steps in the generalized form of the front-door formula ........ 8
Appendix Text S7. Determination of the optimal CVAI cutpoint using maximally selected rank statistics........ 9
Appendix Table S1. Risk of developing MASLD across different levels of a single lifestyle........ 10
Appendix Table S2. Stratification of the risk of developing MASLD by the weighted scores for the four lifestyles........ 11
Appendix Table S3. Descriptive statistics of the complete cases and the proportion of missing ........ 12
Appendix Table S4. Results of the sensitivity analyses........ 14
Appendix Figure S1. The directed acyclic graph of this study........ 15
Appendix Figure S2. Causal diagram for the plausible relations between NWVO, lifestyle, and MASLD in the presence of measured and unmeasured confounders. ...16
Appendix Figure S3. Kaplan-Meier curves grouped by the optimal cutoff value of CVAI ........ 18

**Appendix Text S1. Development of CVAI**

Males: $\text{CVAI} = -267.93 + 0.68 \times \text{Age} + 0.03 \times \text{BMI} + 4.00 \times \text{WC} + 22.00 \times \log_{10} \text{TG} - 16.32 \times \text{HDL-C}$

Females: $\text{CVAI} = -187.32 + 1.71 \times \text{Age} + 4.23 \times \text{BMI} + 1.12 \times \text{WC} + 39.76 \times \log_{10} \text{TG} - 11.66 \times \text{HDL-C}$

Note: CVAI = Chinese visceral adiposity index; BMI = Body mass index; WC = Waist circumference; TG = Triglyceride; HDL-C = High-density lipoprotein cholesterol

**Appendix Text S2. Collection for lifestyle variables**

This study adhered to the *National Basic Public Health Service Specification* established by the National Health Commission of the People's Republic of China for the collection of lifestyle variables. These variables were investigated face-to-face by trained primary care physicians for quantitative data and transformed into qualitative data to establish health profiles and follow-up records. The quantitative data obtained from the survey were then utilized to ascertain the lifestyle effectiveness following Chinese guidelines for the management of hypertension and diabetes, as follows:

(1) Effective exercise: A moderate-intensity exercise session was defined as effective if it lasted more than 30 minutes. The intensity of exercise was determined by the subjective sensation of exertion as perceived by the respondents. The general feeling associated with moderate-intensity exercise is one of heart rate acceleration, slight sweating, and slight fatigue, or the ability to articulate complete sentences during moderate-intensity exercise, but not to sing.

(2) Effective drinking: The respondents were surveyed about the type and amount of alcoholic beverages they consumed, and this was converted into an alcohol intake. The daily intake of alcohol that is defined as effective drinking is a quantity that exceeds 25 grams for males and 15 grams for females.

(3) Effective smoking: Active daily consumption of tobacco products is defined as effective smoking, irrespective of the quantity of tobacco consumed. Previous tobacco consumption among quitters, as well as exposure to second-hand smoke among the respondents, could not be investigated.

(4) Effective salt intake: The daily salt intake of the respondents was obtained from dietary surveys. Low effective salt intake is defined as less than 3 grams of sodium salt per day, and high effective salt intake is defined as a daily sodium salt intake of greater than 3 grams.

**Appendix Text S3. Weighted score for the four lifestyles**

The causal relationship between a single lifestyle and the risk of developing MASLD among participants was assessed using marginal structural models. This approach is based on a causal inference framework, whereby generalized propensity scores are calculated by regressing lifestyle estimates against potential confounding variables. Subsequently, stabilized weights are established based on generalized propensity scores to simulate randomized controlled trials, thereby creating a pseudo-population. The potential confounding effects that may arise in this pseudo-population have been effectively balanced by inverse probability of treatment weighting, and it is therefore believed that the observed data will generate causal insights.

The weighted score was derived for each individual using the following formula:

$$\text{Weighted score for the four lifestyles } = \sum_{n=1\cdots k} \beta_n X_n$$

where $X$ is the value of an individual level of lifestyle and $\beta$ is the natural logarithm of the hazard ratio for developing metabolic dysfunction-associated steatotic liver disease for the specific level of lifestyle. The effect size of each level of lifestyle ($\beta$) was derived from the marginal structural models. **(Appendix Table S1)**

A total of 144 potential combinations of the four lifestyles were enumerated using the exhaustive method, with weighted scores ranging from 0 to 3.861. The weighted scores were divided into four intervals based on quartiles to represent different levels of an overall healthy lifestyle, with higher scores indicating a more stringent healthy lifestyle. It was found that individuals with weighted scores below the $75^{th}$ percentile might benefit from risk reduction by a small amount. Notably, an over 90% reduction in the risk of developing MASLD was observed when the weighted score exceeded the $75^{th}$ percentile. **(Appendix Table S2)**

**Appendix Text S4. Diagnostic criteria of MASLD**

MASLD was defined as the presence of steatotic liver disease combined with at least 1 of the 5 following cardiometabolic adult criteria: (1) Body mass index ≥25 $kg/m^2$ [23 Asia] OR waist circumference >94 cm (M) 80 cm (F) OR ethnicity adjusted equivalent, (2) Fasting serum glucose >5.6 mmol/L [100 mg/dl] OR2-hour post-load glucose levels ≥7.8 mmol/L [≥140 mg/dl] OR HbA1c >5.7% [39 mmol/L] OR type 2 diabetes OR treatment for type 2 diabetes, (3) Blood pressure >130/85 mmHg OR specific antihypertensive drug treatment, (4) Plasma triglycerides ≥1.70 mmol/L [150 mg/dl] OR lipid lowering treatment, and (5) Plasma high-density lipoprotein cholesterol ≤1.0 mmol/L [40 mg/dl] (M) and ≤1.3 mmol/L [50 mg/dl] (F) OR lipid lowering treatment.

**Appendix Text S5. Assumptions of the generalized front-door formula and their justification**

The validity of the generalized front-door approach rests on three key assumptions:

First, the mediator (unhealthy lifestyle) must block all paths from the exposure (NWVO) to the outcome (MASLD) that are subject to unmeasured confounding. Formally, this requires that any unmeasured confounder affecting both exposure and outcome must exert its influence exclusively through the mediator. In our setting, this assumption implies that if unmeasured factors (e.g., genetic predisposition, early-life metabolic programming) influence both NWVO and MASLD, their effects are at least partially captured by differences in lifestyle patterns. While this assumption is non-testable, we argue its plausibility because lifestyle encompasses multiple behavioral domains (physical activity, diet, smoking, alcohol) that are well-established determinants of both visceral adiposity and liver disease, thereby serving as a comprehensive conduit for many upstream confounders. Moreover, our sensitivity analyses using alternative lifestyle definitions and stable NWVO subgroups provide indirect evidence of robustness to potential violations.

Second, there is no unmeasured confounding of the exposure–mediator relationship. That is, after adjusting for the covariates identified by the directed acyclic graph (sex, age, education, marital status, follow-up results for hypertension and diabetes, and drug compliance), no unmeasured variables confound the association between NWVO and lifestyle. We minimized this risk by leveraging the comprehensive NBPHSP dataset, which includes detailed sociodemographic and clinical information relevant to both NWVO and lifestyle behaviors.

Third, there is no unmeasured confounding of the mediator–outcome relationship. This requires that, conditional on the exposure (NWVO) and the same set of covariates, no unmeasured variables confound the association between lifestyle and MASLD. We addressed this through the same covariate adjustment strategy, and further support this assumption by noting that all lifestyle factors included (physical activity, alcohol,

smoking, salt intake) are established risk factors for MASLD whose effects are well-characterized in the literature, reducing the likelihood of major unmeasured confounders specific to this pathway.

In addition to these structural assumptions, the front-door formula requires that the mediator is measured without error and that the mediator–outcome relationship is correctly specified. We addressed the former by using standardized assessment protocols within the NBPHSP; the latter was evaluated by comparing model specifications in sensitivity analyses, which yielded consistent results.

**Appendix Text S6. Steps in the generalized form of the front-door formula**

**Notation:** $X =$ Exposure (binary; $x$ and $x^*$), $M =$ Mediator (binary), $Y =$ Outcome (binary), $C =$ Measured confounders, $X' =$ using $X$ for confounding adjustment at the $Y$-stage of the front-door formula, $U =$ uncontrolled confounders between $X$ and $Y$.

$E(Y_{Mx}) = E(Y_{X'Mx}) = \Sigma_{m,c} P(M = m|X = x, C = c) \Sigma_{X'} E(Y|M = m, X' = x', C = c) P(X' = x', C = c)$

Path-specific front-door effect (PSFDE): the contrast between $E(Y_{Mx})$ and $E(Y_{Mx^*})$, with $E(Y_{Mx}) = E(Y_{X'Mx}) = \sum_{x'} E(Y_{x'Mx}) P(x') = E(Y_{xMx}) P(x) + E(Y_{x^*Mx}) P(x^*)$, where equality holds if no *U-M* interaction.

**Step 1:** Obtain Empirical Parameters.

**Step 1a.** Model for the mediator given the exposure and the confounders:

$$P(M = m|X = x, C = c)$$

**Step 1b.** Model for the outcome given the exposure, the mediator, and the confounders:

$$E(Y|M = m, X' = x', C = c)$$

**Step 2:** Simulate the Potential Mediator and the Potential Outcome.

**Step 2a.** Create two copies of the original sample.

**Step 2b.** Simulate the exposure variable that is marginally independent of the confounders.

**Step 2c.** Simulate the mediator variable as a function of its parents (the simulated exposure in step 2b and the confounders) using empirical parameters obtained in step 1a.

**Step 2d.** Simulate the outcome variable as a function of its parents (the original exposure [not the simulated exposure in step 2b], the simulated mediator in step 2c, and the confounders) using empirical parameters obtained in step 1b.

**Step 3:** Fit the Final Marginal Structural Model.

Regress the simulated outcome in step 2d on the simulated exposure in step 2b to obtain point estimates of the marginal effect using the pooled data with two copies of the original sample.

**Appendix Text S7. Determination of the optimal CVAI cutpoint using maximally selected rank statistics**

The optimal cutoff value of the CVAI for predicting MASLD risk was identified using maximally selected rank statistics. This method systematically evaluates all potential cutpoints for a continuous variable to determine the threshold that maximizes the separation between two groups for the time-to-event outcome.

The procedure is based on computing a rank-order test statistic for every possible dichotomization of the cohort defined by a candidate cutpoint $C$. Specifically, for each value $C$, the sample is split into two subsets: one with CVAI $\leq C$ and the other with CVAI $> C$. The most prognostic cutpoint is selected as the value that maximizes the standardized log-rank statistic across all allowable splits, thereby identifying the point with the strongest association with the outcome.

To account for the inherent multiple testing problem, a modified $P$-value was calculated using a standard procedure that incorporates a correction based on the asymptotic distribution of the maximum selected statistic. This widely adopted approach ensures the statistical robustness of the identified threshold by controlling for overoptimism. Furthermore, a constraint was applied to require that each subgroup defined by the cutpoint contain at least 30% of the observations, guaranteeing sufficient sample size in both groups and improving the clinical interpretability and stability of the result.

This method is well-suited for identifying clinically meaningful thresholds in continuous variables without requiring prior assumptions about their distribution or functional form, making it an objective and powerful tool for exploratory cutpoint determination in the present study.

**Appendix Table S1. Risk of developing MASLD across different levels of a single lifestyle**

| Variable | $\beta$ | Standard Error | *P*-value |
|---|---|---|---|
| **Exercise frequency** | | | |
| Never | | Reference | |
| Regular weekly | -0.341 | 0.045 | <0.001 |
| Occasionally | -0.383 | 0.094 | <0.001 |
| Daily | -0.483 | 0.267 | 0.071 |
| **Alcohol consumption** | | | |
| Everyday | | Reference | |
| Often | -0.224 | 0.063 | <0.001 |
| Occasionally | -0.395 | 0.169 | 0.020 |
| Never | -1.441 | 0.477 | 0.003 |
| **Smoking status** | | | |
| Current | | Reference | |
| Ever | -0.375 | 0.063 | <0.001 |
| Never | -1.880 | 0.230 | <0.001 |
| **Salt intake** | | | |
| High | | Reference | |
| Low | -0.057 | 0.014 | <0.001 |

**Appendix Table S2. Stratification of the risk of developing MASLD by the weighted scores for the four lifestyles**

| Percentile interval | $\beta$ | Standard Error | $P$-value |
|---|---|---|---|
| 0-25% | | Reference | |
| 25%-50% | -0.049 | 0.028 | 0.077 |
| 50%-75% | -0.292 | 0.037 | <0.001 |
| 75%-100% | -2.333 | 0.201 | <0.001 |

Notes: Quartiles of weighted scores are 0 ($0^{th}$), 0.762 ($25^{th}$), 1.375 ($50^{th}$), 2.323 ($75^{th}$), and 3.861 ($100^{th}$).

**Appendix Table S3. Descriptive statistics of the complete cases and the proportion of missing**

| | Total ($N$ = 26289) | Complete cases ($N$ = 20404) | Missing $n$ (%) |
|---|---|---|---|
| **Sex, Females (%)** | 10975 (41.7) | 8399 (41.2) | 0 (0) |
| **Age group (%)** | | | 0 (0) |
| 50 to 59 years | 2370 (9.0) | 1874 (9.2) | |
| 60 to 69 years | 16448 (62.6) | 12660 (62.0) | |
| 70 to 79 years | 6251 (23.8) | 4900 (24.0) | |
| 80 to 89 years | 1148 (4.4) | 919 (4.5) | |
| 90 years and older | 72 (0.3) | 51 (0.2) | |
| **Education (%)** | | | 4 (0.01) |
| College or above | 4379 (16.7) | 3410 (16.7) | |
| High school | 6970 (26.5) | 5396 (26.4) | |
| Second school | 6662 (25.3) | 5161 (25.3) | |
| Primary school | 7120 (27.1) | 5546 (27.2) | |
| Illiterate or semiliterate | 1158 (4.4) | 891 (4.4) | |
| **Marriage (%)** | | | 9 (0.03) |
| Never | 17 (0.1) | 13 (0.1) | |
| Married | 25203 (95.9) | 19569 (95.9) | |
| Widowed | 977 (3.7) | 753 (3.7) | |
| Divorce | 92 (0.3) | 69 (0.3) | |
| **BMI (mean, SD)** | 22.66 (1.97) | 22.73 (2.04) | 465 (1.76) |
| **Waist, cm (mean, SD)** | 83.19 (5.80) | 83.89 (6.18) | 660 (2.51) |
| **TG, mmol/L (mean, SD)** | 1.38 (0.55) | 1.38 (0.57) | 1227 (4.67) |
| **HDL-C, mmol/L (mean, SD)** | 1.27 (0.26) | 1.28 (0.27) | 900 (3.42) |
| **Follow-up result for hypertension, Unsatisfactory control (%)** | 1481 (5.6) | 1235 (6.1) | 2271 (8.64) |
| **Follow-up result for diabetes, Unsatisfactory control (%)** | 4238 (16.1) | 3469 (17.0) | 2018 (7.68) |
| **Drug compliance for hypertension (%)** | | | 2297 (8.74) |
| Regularly | 23802 (90.5) | 18515 (90.7) | |
| Intermittently | 940 (3.6) | 686 (3.4) | |
| Not taking medicine | 1546 (5.9) | 1200 (5.9) | |
| No medication required | 1 (0.0) | 3 (0.0) | |
| **Drug compliance for diabetes (%)** | | | 2011 (7.68) |
| Regularly | 23486 (89.3) | 18153 (89.0) | |
| Intermittently | 833 (3.2) | 640 (3.1) | |
| Not taking medicine | 1723 (6.6) | 1475 (7.2) | |
| No medication required | 247 (0.9) | 136 (0.7) | |
| **Exercise frequency (%)** | | | 11 (0.04) |

| | | | |
|---|---|---|---|
| Daily | 14755 (56.1) | 11556 (56.6) | |
| Regular weekly | 5490 (20.9) | 4265 (20.9) | |
| Occasionally | 2256 (8.6) | 1729 (8.5) | |
| Never | 3788 (14.4) | 2854 (14.0) | |
| **Alcohol consumption (%)** | | | 43 (0.16) |
| Never | 22520 (85.7) | 17458 (85.6) | |
| Occasionally | 2449 (9.3) | 1899 (9.3) | |
| Often | 615 (2.3) | 481 (2.4) | |
| Everyday | 705 (2.7) | 566 (2.8) | |
| **Smoking status (%)** | | | 19 (0.07) |
| Never | 22667 (86.2) | 17364 (85.1) | |
| Ever | 1768 (6.7) | 1466 (7.2) | |
| Current | 1854 (7.1) | 1574 (7.7) | |
| **Salt intake, Low (%)** | 13887 (52.8) | 10983 (53.8) | 2546 (9.68) |

Notes: BMI = Body mass index; TG = Triglyceride; HDL-C = High-density lipoprotein cholesterol

**Appendix Table S4. Results of the sensitivity analyses**

| Analysis Scenario | Exposure to Mediator (Adjusted *HR*, 95% *CI*) | Mediator to Outcome (Adjusted *HR*, 95% *CI*) | Natural indirect effects (Adjusted *HR*, 95% *CI*) | Mediating proportion (%, 95% *CI*) |
|---|---|---|---|---|
| Alternative CVAI cutoff | 2.101 (2.015, 2.190) | 1.108 (1.084, 1.133) | 1.298 (1.021, 1.651) | 16.452 (10.123, 28.665) |
| Alternative lifestyle cutoff | 1.682 (1.601, 1.767) | 1.063 (1.042, 1.084) | 1.205 (1.073, 1.353) | 18.151 (11.251, 30.112) |
| Sex-stratified: Females | 2.383 (2.275, 2.496) | 1.124 (1.087, 1.162) | 1.451 (1.078, 1.953) | 21.034 (12.487, 35.624) |
| Sex-stratified: Males | 2.148 (1.823, 2.537) | 1.098 (1.052, 1.146) | 1.318 (0.892, 1.957) | 16.758 (7.234, 34.521) |
| Complete-case analysis | 2.355 (2.248, 2.468) | 1.115 (1.086, 1.144) | 1.409 (1.023, 1.942) | 19.554 (11.892, 33.987) |
| Stable NWVO subgroup | 2.451 (2.321, 2.588) | 1.121 (1.090, 1.153) | 1.501 (1.152, 1.956) | 22.448 (14.912, 36.812) |

Notes: CVAI = Chinese visceral adiposity index; NWVO = Normal-weight visceral obesity; HR = Hazard ratio; CI = Confidence interval

**Appendix Figure S1. The directed acyclic graph of this study**

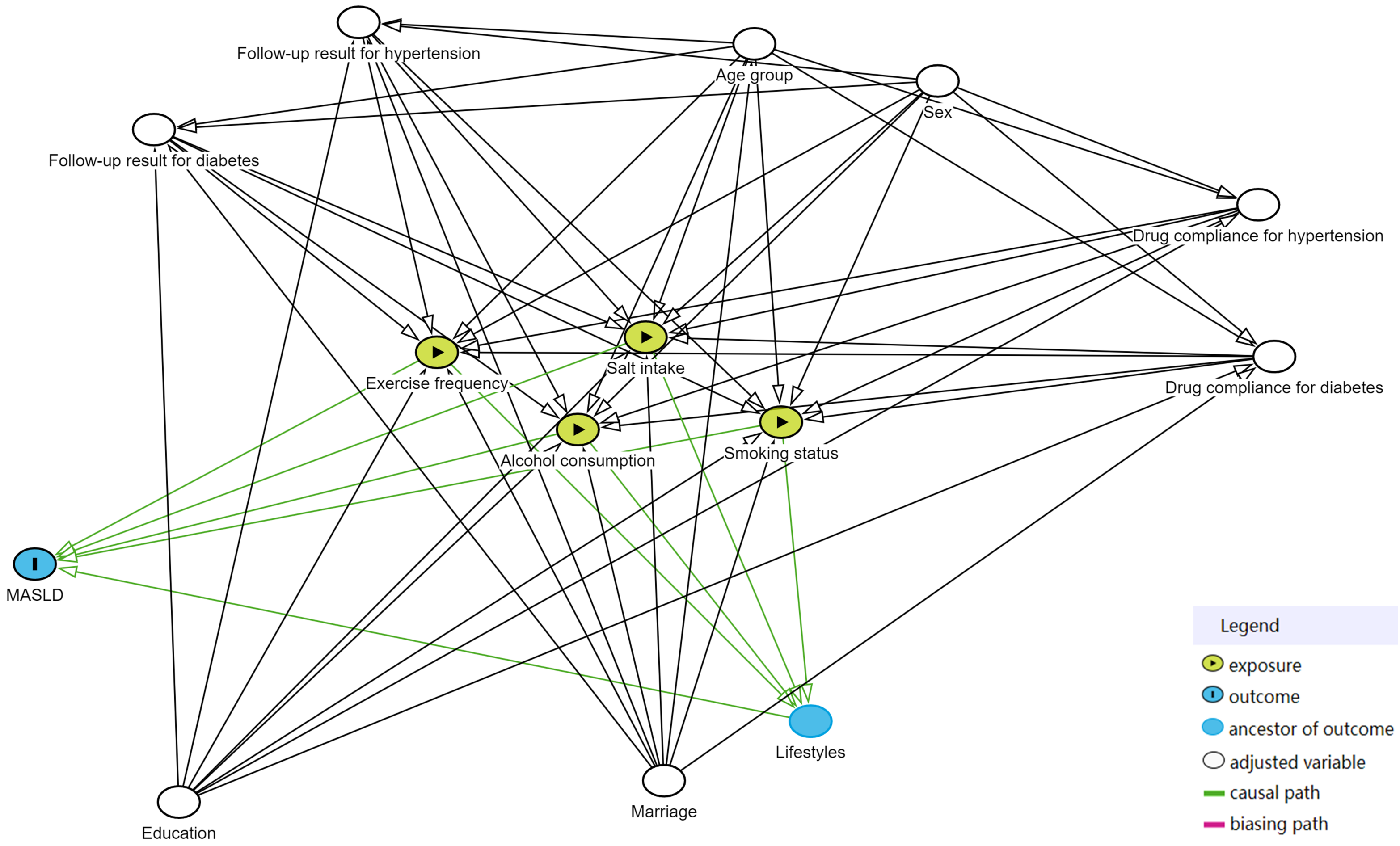

**Appendix Figure S2. Causal diagram for the plausible relations between NWVO, lifestyle, and MASLD in the presence of measured and unmeasured confounders.**

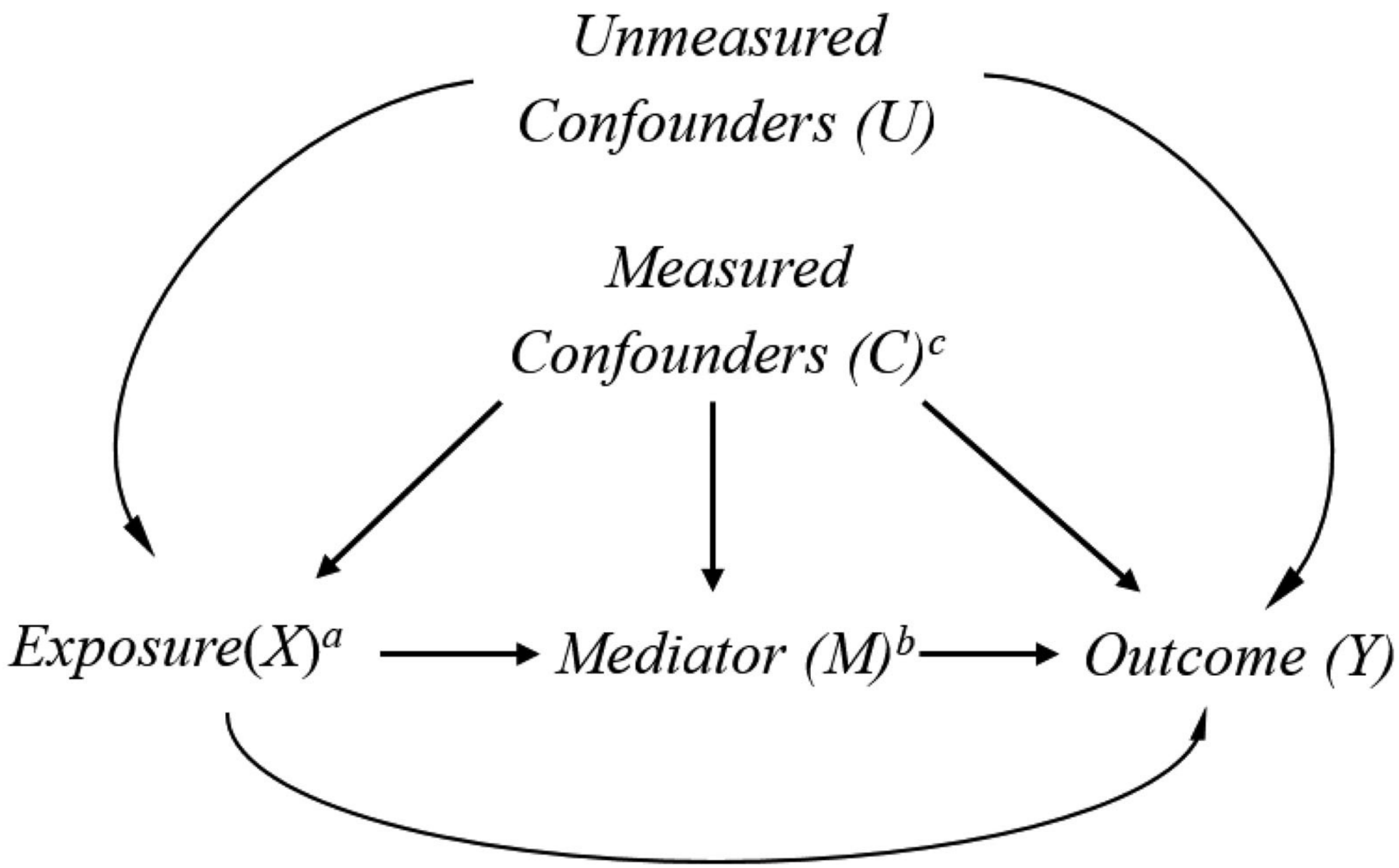


Notes: [a]Normal-weight visceral obesity (X) was defined using the Chinese Visceral Adiposity Index (CVAI ≥ 79.19) alongside normal body size criteria (BMI < 24 kg/$m^2$ and waist circumference < 90 cm for males or < 85 cm for females). [b]Unhealthy lifestyle (M) was defined as a composite lifestyle-weighted score ≤ 2.323 (≤ 75th percentile), derived from four dimensions: exercise frequency, alcohol consumption, smoking status, and

dietary salt intake. [c]Metabolic dysfunction-associated steatotic liver disease (Y) was diagnosed by transabdominal ultrasound and cardiometabolic criteria. [d]Measured covariates (C) included sex, age group, education, marital status, follow-up results for hypertension and diabetes, and drug compliance for hypertension and diabetes. [e]U represents unmeasured confounders between exposure and outcome (e.g., genetic predisposition, early-life metabolic programming) that the generalized front-door formula is specifically designed to accommodate. The three structural conditions required for valid estimation are: (1) the mediator intercepts all paths from X to Y that are influenced by U; (2) no unmeasured confounding of the X→M relationship after adjusting for C; and (3) no unmeasured confounding of the M→Y relationship after adjusting for C and X. The path-specific front-door effect (X→M→Y) is estimated through a simulation-based procedure detailed in **Appendix Text S6**.

**Appendix Figure S3. Kaplan-Meier curves grouped by the optimal cutoff value of CVAI**

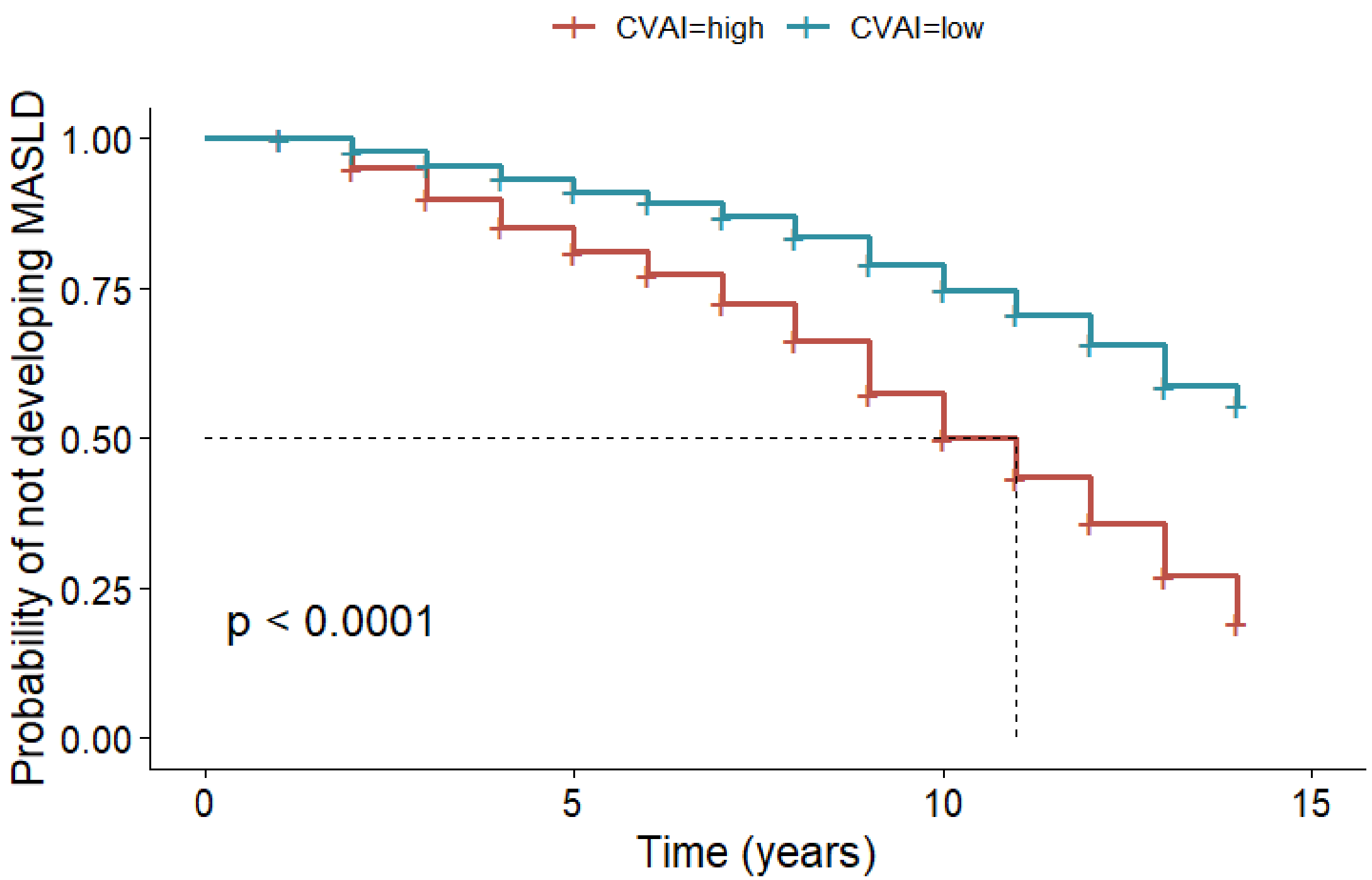


Notes: CVAI = Chinese visceral adiposity index; "CVAI = high" means CAVI >131.35; "CVAI = low" means CAVI ≤131.35
